# Gastrointestinal/genitourinary adverse event after intensity modulated versus three-dimensional primary radiation therapy in the treatment of prostate cancer: systematic review and meta-analysis

**DOI:** 10.7150/jca.87626

**Published:** 2023-09-11

**Authors:** Wei Guo, Yun-Chuan Sun, Li-Yuan Zhang, Xiao-Ming Yin

**Affiliations:** 1 Department of Radiation Oncology, Hebei Province Cangzhou Hospital of Integrated Traditional and Western Medicine, Cangzhou, Hebei, 061000, China.; 2 Key Laboratory of Endocrinology of National Health Commission, Department of Endocrinology, State Key Laboratory of Complex Severe and Rare Diseases, Peking Union Medical College Hospital, Chinese Academy of Medical Science and Peking Union Medical College, Beijing, 100730, China.

**Keywords:** IMRT, 3D-CRT, Prostate cancer, Efficacy, Adverse event

## Abstract

**Objective:** Prostate cancer (PCa) is one of the most common cancers in the world. The potential benefits of intensity modulated radiation therapy (IMRT) over three-dimensional conformal radiation therapy (3D-CRT) for PCa primary radiation therapy treatment have not yet been clarified. Therefore, this meta-analysis was conducted to assess whether IMRT could improve clinical outcomes in comparison with 3D-CRT in patients diagnosed with PCa.

**Materials and methods:** Relevant studies were identified through searching related databases till December, 2022. Hazard ratio (HR) or risk ratio (RR) with its corresponding 95% confidence interval (CI) was used as pooled statistics for all analyses.

**Results:** The incidence of grade 2 or worse acute adverse gastrointestinal (GI) event was analyzed and the pooled data revealed a clear decreasing trend in the IMRT compared with 3D-CRT (RR=0.62, 95% CI: 0.45-0.84, *p*=0.002). IMRT slightly increased the grade ≥ 2 acute genitourinary (GU) adverse event in comparison with the 3D-CRT (RR=1.10, 95% CI: 1.02-1.19, *p*=0.015). The IMRT and the 3D-CRT of patients showed no substantial differences in grade ≥ 2 late GI adverse event (RR =0.62, 95% CI: 0.36-1.09, *p*=0.1). In those included studies, there was no significant difference between IMRT and 3D-CRT in grade 2-4 late GU adverse event (RR =1.08, 95% CI: 0.77-1.51, *p*=0.65). There was a significant difference in biochemical control favoring IMRT (RR =1.13, 95% CI: 1.05-1.22, *p*=0.002). IMRT showed modest increase in biochemical control in comparison with 3D-CRT.

**Conclusion:** In general, based on the above results, IMRT should be considered as a better choice for the treatment of PCa. More randomized controlled trials are needed to determine the subset of patients diagnosed with PCa.

## Introduction

Prostate cancer (PCa) is among the most prevalent neoplastic diseases worldwide, particularly in Northern America and Western Europe 1. According to the outcomes of prior investigations, radiation therapy (RT) has gained widespread employment in the treatment of PCa 2, 3. Several clinical studies have found that increasing the dose is related with better biochemical and overall survival results 4-7. As the majority of individuals with non-metastatic PCa can endure for over a decade, it is imperative to opt for RT techniques that reduce RT-associated toxicity to enhance their standard of life 8, 9. Enhanced dosages, on the other hand, may cause enhanced normal tissue toxicity, which includes delayed gastrointestinal (GI) and genitourinary (GU) damage 10.

New RT procedures have arisen as a result of the development of enhanced radiation technologies and have been used in clinical settings. The delivery of a radiation dose that conforms to the target volume of the tumor is made easier by three-dimensional conformal radiation therapy (3D-CRT). 11. Consequently, the target dose is greatly increased while the impact on normal tissue is simultaneously diminished 12. The most sophisticated kind of 3D-CRT, intensity modulated radiation therapy (IMRT), which generates non-uniform fields to augment the radiation dosage supplied to the intended target volume while limiting irradiation to the organs at danger, represents the culmination of the development of RT techniques 13. However, the likelihood of a minor miss represents a potential shortcoming of IMRT. When using IMRT, it is also necessary to take into account the dose homogeneity, the escalation of irradiation doses to bigger volumes of healthy tissues, and longer planning timeframes 14. The amplified total body exposure and monitor units increase the likelihood of second malignancies when utilizing IMRT, as opposed to conventional RT 15.

Nonetheless, the prospective advantages of IMRT over 3D-CRT for first radiation therapy for treating PCa have not been proven. As a result, this meta-analysis was carried out to determine whether IMRT may improve clinical results in patients with PCa when compared to 3D-CRT.

## Materials and methods

### Literature search

The Preferred Reporting Items for Systematic Reviews and Meta-analyses (PRISMA) criteria were followed in this meta-analysis 16. Up until December 2022, we did a literature search utilizing the databases Pubmed, Embase, and Web of Science. The following keywords were used in the search strategy: "prostate cancer [Title/Abstract]", "intensity modulated radiation therapy [Title/Abstract]", "IMRT [Title/Abstract]", "three-dimensional conformal radiation therapy [Title/Abstract]", and "3D-CRT [Title/Abstract]". In addition, we looked at abstracts from major academic conferences. To find possibly eligible articles, the references of the included studies were also evaluated.

### Study selection

The selected studies were required to meet the following eligibility criteria: a) all patients were confirmed to have PCa histologically and had not undergone radical prostatectomy; b) the study had clearly defined case selection criteria; c) interventions primarily focused on IMRT and 3D-CRT, and both techniques were conducted within the same study; d) essential data for hazard ratio (HR) or risk ratio (RR) with its corresponding 95% confidence interval (CI) was reported or could be calculated utilizing Tierney's method 17; e) published in full-text form; f) published in English; g) single prescription dose less than 2.5Gy. Studies were excluded if they fulfilled any of the following criteria: a) insufficient data to estimate HRs or RRs with 95% CIs; b) patients who had undergone pelvic irradiation or radical prostatectomy; c) animal experiments; d) letters, meeting abstracts, or review papers; and e) not presented in English.

### Quality assessment of publications

The Newcastle-Ottawa quality assessment scale (NOS) was used in the investigation to rate the effectiveness of both cohort and case-control studies 18. The NOS scale has three components: defining and selecting case and control groups, comparing case and control groups, and determining exposure. The utmost score is 9 points. In this study, a score of ≥ 7 is established as high-quality research, 4-6 is categorized as medium quality research, and ≤ 3 is characterized as low-quality research. As for the randomized controlled study, it was conducted utilizing the Jadad scale for quality evaluation. The method of creating random grouping sequences, the double-blind procedure, withdrawal, and lack of follow-up are among the grading factors. The total score is 5, with 1-2 indicating bad quality and 3-5 indicating high quality 19.

### Data extraction

Two investigators, W.G. and L.Y.Z., used a predetermined process to collect data from the eligible studies. The extracted data included the first author's name, year of publication, study design, duration of follow-up, patient count, radiation dose, planning target volume (PTV), TNM classification, scoring criteria, and androgen deprivation therapy (ADT) details, as well as their corresponding outcomes. For scoring of acute and late radiation damage, Common Terminology Criteria of Adverse Events (CTCAE) or Radiation Therapy Oncology Group (RTOG) Common Toxicity Criteria are typically used. Any inconsistencies or disagreements between the two reviewers were resolved with the help of a third investigator, Y.C.S.

### Statistical analysis

The HRs and RRs for clinical results were collected directly from each trial if available, or estimated from raw data using the approach described by Tierney et al. 17. Cochran's Q test and the Higgins I-squared statistic were used to assess the heterogeneity of the pooled results. The random-effects model was used if the I-squared statistic was larger than 50% and the P-value for heterogeneity was less than 0.1, suggesting significant heterogeneity; otherwise, the fixed-effects model was used. Additionally, sensitivity analyses were carried out to assess the effect of particular research on the total estimate. Begg's funnel plot was also evaluated for possible publication bias. STATA 12.0 software (Stata Corp, College Station, TX, USA) was used for the statistical analysis. A P-value of 0.05 or less was judged to be statistically noteworthy.

## Results

### Literature search and summary of studies

**Figure 1:**
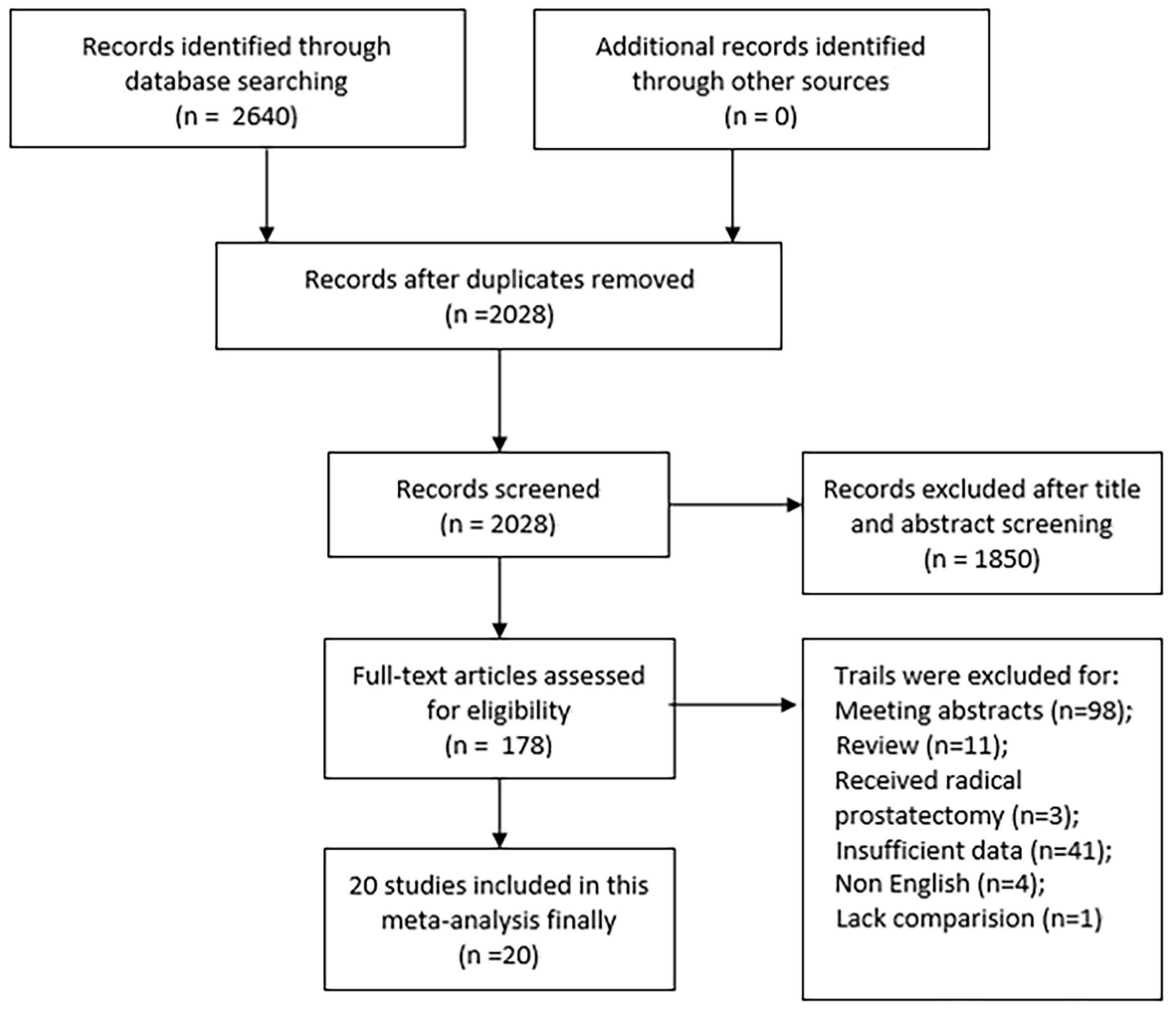
Flow chart of the included trials.

Initially, the searched keywords yielded an overall number of 2640 articles. After deleting duplicates, 2028 articles remained, of which 1850 were removed after screening titles and abstracts. Following that, full texts and data integrity were thoroughly checked, resulting in the exclusion of an additional 158 studies. Finally, a total of 20 papers were judged eligible and included in the final meta-analysis 20-39. **Figure 1** depicts the selection procedure for our articles. The Jadad scale was used to evaluate randomized controlled trials, with all scores equal to or greater than 3 meeting the inclusion requirements. The remaining retrospective investigations were evaluated using the NOS scale, with all studies scoring 6 or higher (**Supplement Table 1**).

The overall number of patients included in the meta-analysis was 8645, with patient numbers ranging from 27 to 1571 per study. The basic characteristics of the included studies are shown in **Table 1**. In the study design, retrospective cohort studies (n = 13) were more common than prospective cohort studies (n = 4). The primary tumor doses were 70-82 Gy in the IMRT group and 66-81 Gy in the 3D-CRT group. The median duration of follow-up ranged from 3 to 120 months. Twelve of the studies included evaluated the acute adverse event of an IMRT group to that of a 3D-CRT group, including acute GI adverse event (n = 10) and acute GU adverse event (n = 12). Furthermore, ten studies evaluated the late adverse event effects of the IMRT group to those of the 3D-CRT group, including late GI (n = 8) and late GU adverse events (n = 9). Furthermore, seven investigations compared the biochemical control of the IMRT and 3D-CRT groups.

### Acute GI adverse event

**Figure 2:**
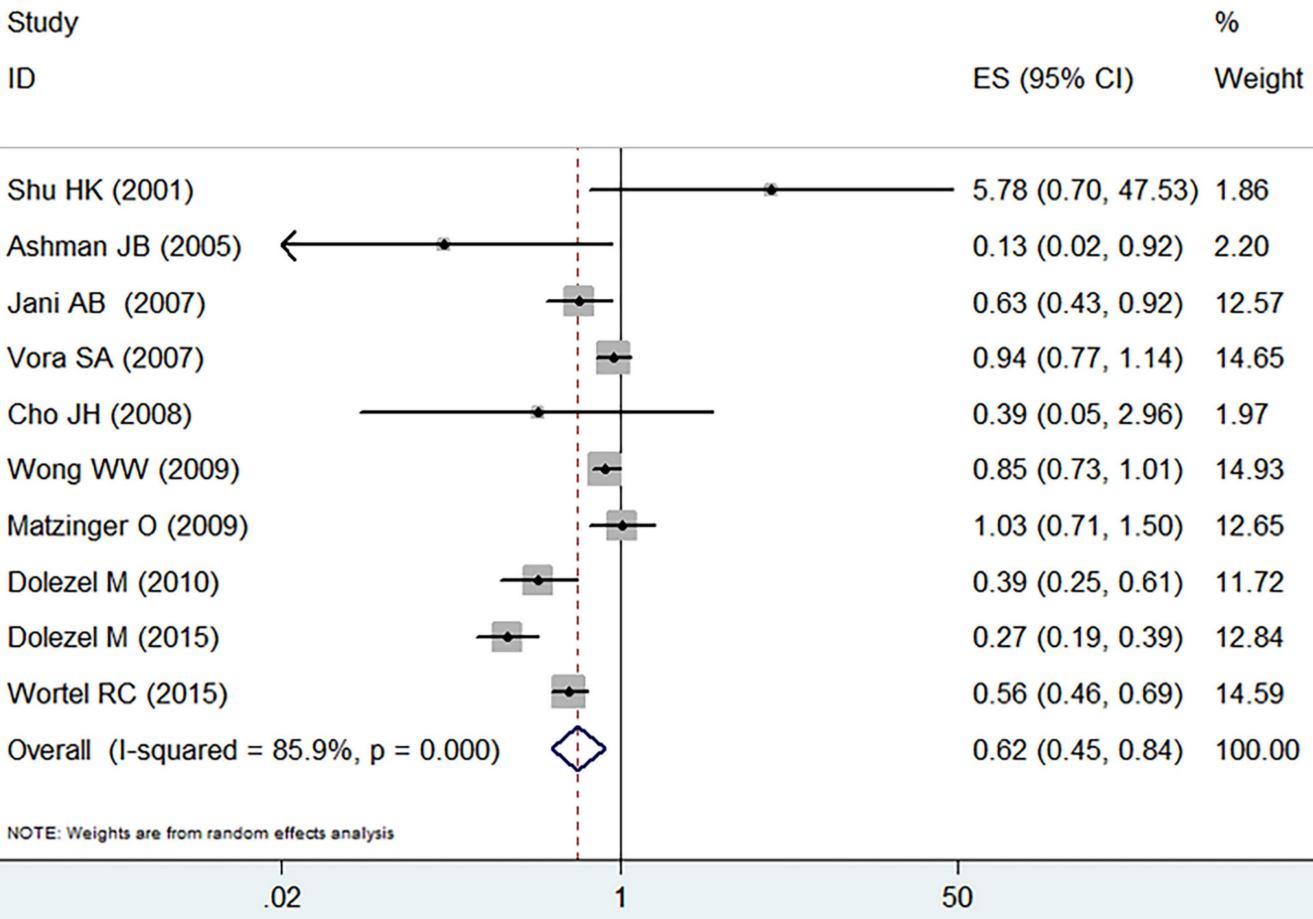
Forest plot for acute GI adverse event.

The incidence of grade 2 or worse acute adverse GI event was analyzed by the random effect model due to heterogeneous outcomes (I^2^=85.9%, *p*=0.000) and the pooled data revealed a clear decreasing trend in the IMRT compared with 3D-CRT (RR=0.62, 95% CI: 0.45-0.84, *p*=0.002,** Figure 2**).

### Acute GU adverse event

**Figure 3:**
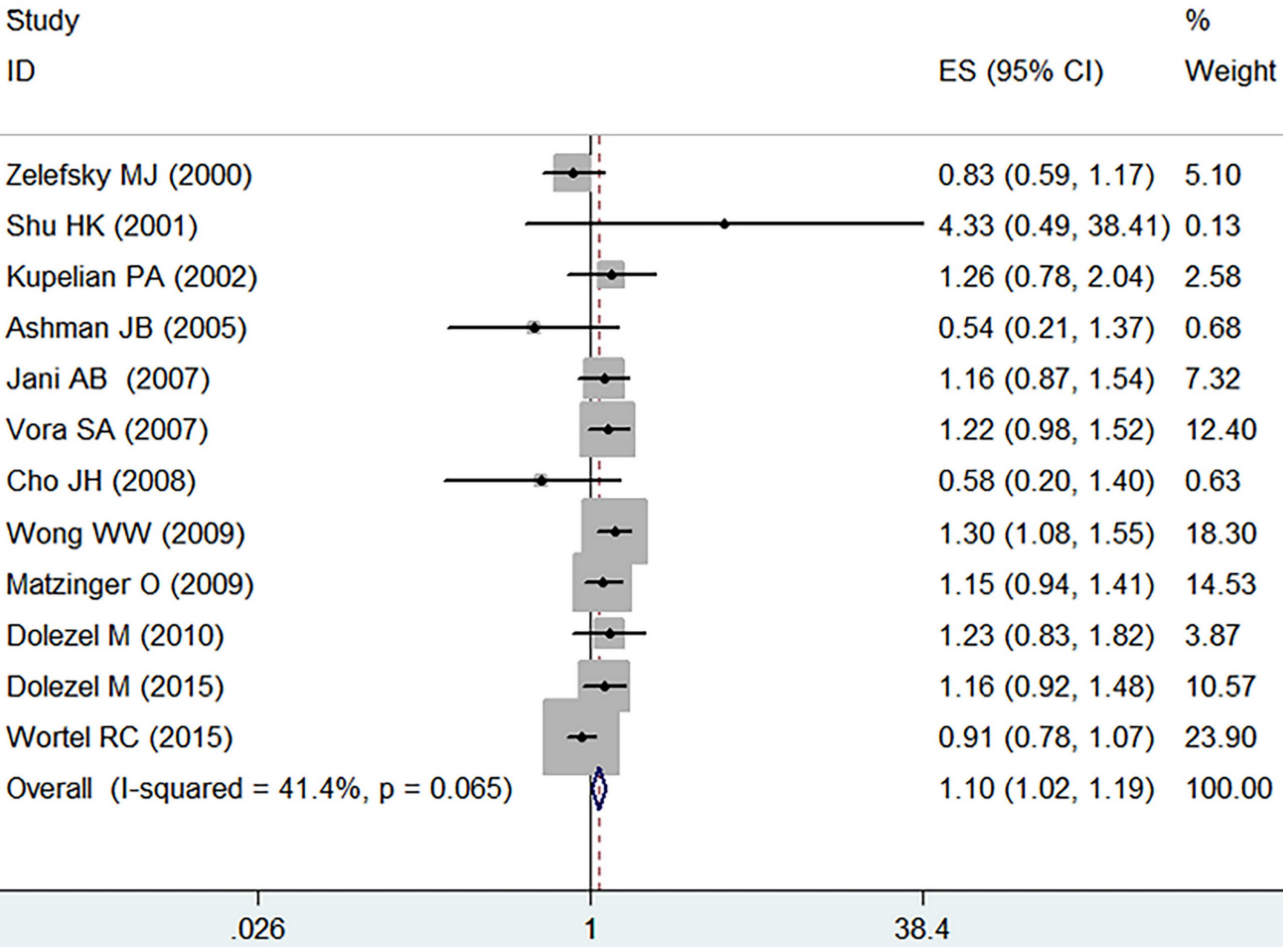
Forest plot for acute GU adverse event.

Analysis by the fixed-effect model (I^2^=41.4%, *p*=0.065) showed that IMRT slightly increased the grade ≥ 2 acute GU adverse event in comparison with the 3D-CRT (RR=1.10, 95% CI: 1.02-1.19, *p*=0.015, **Figure 3**).

### Late GI adverse event

**Figure 4:**
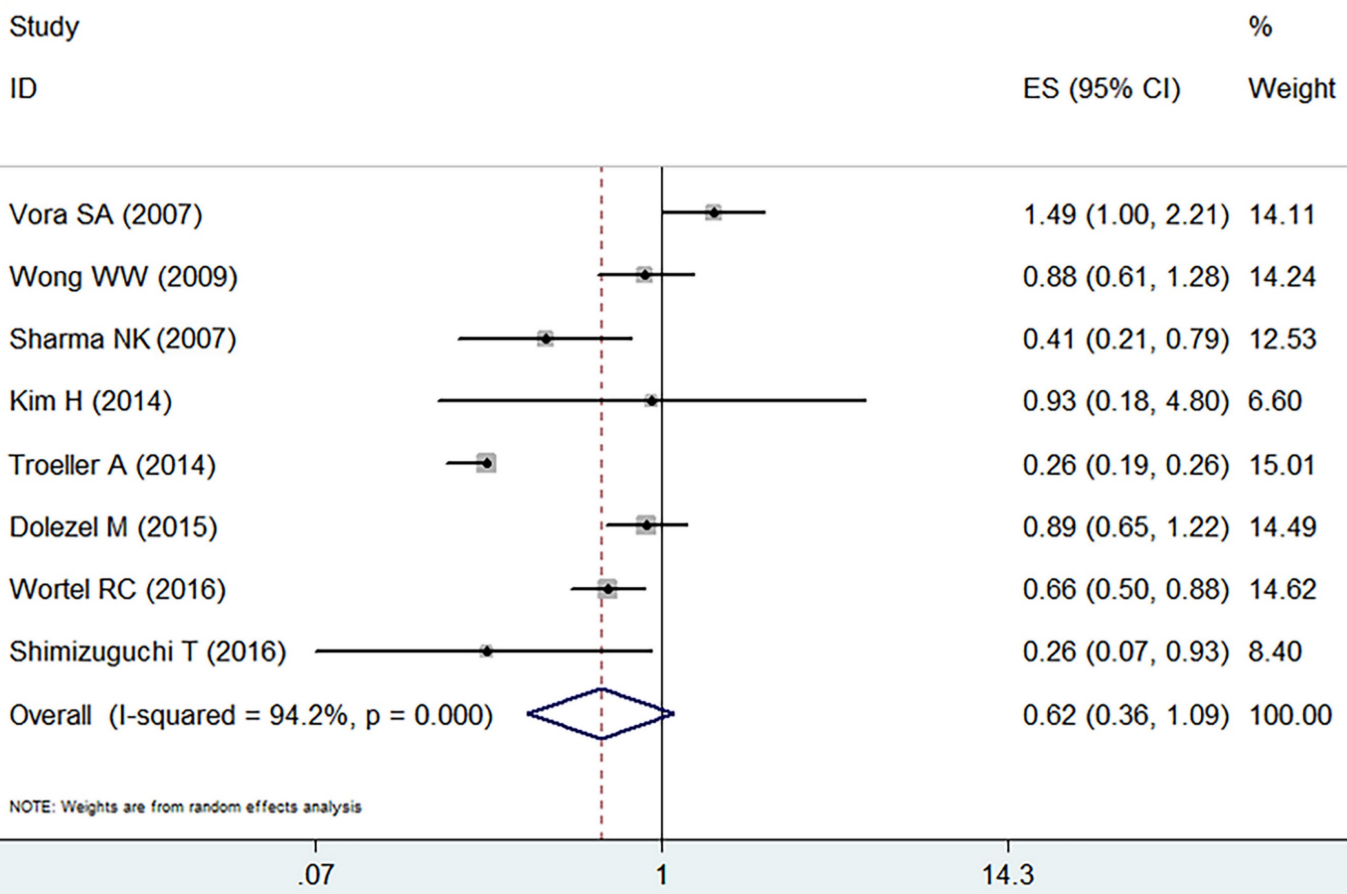
Forest plot for late GI adverse event.

The IMRT and the 3D-CRT of patients showed no substantial differences in grade ≥ 2 late GI adverse event (RR =0.62, 95% CI: 0.36-1.09, *p*=0.1,** Figure 4**) and showed a high level of heterogeneity based on the random effect model (I^2^=94.2%, *p*=0.000).

### Late GU adverse event

**Figure 5:**
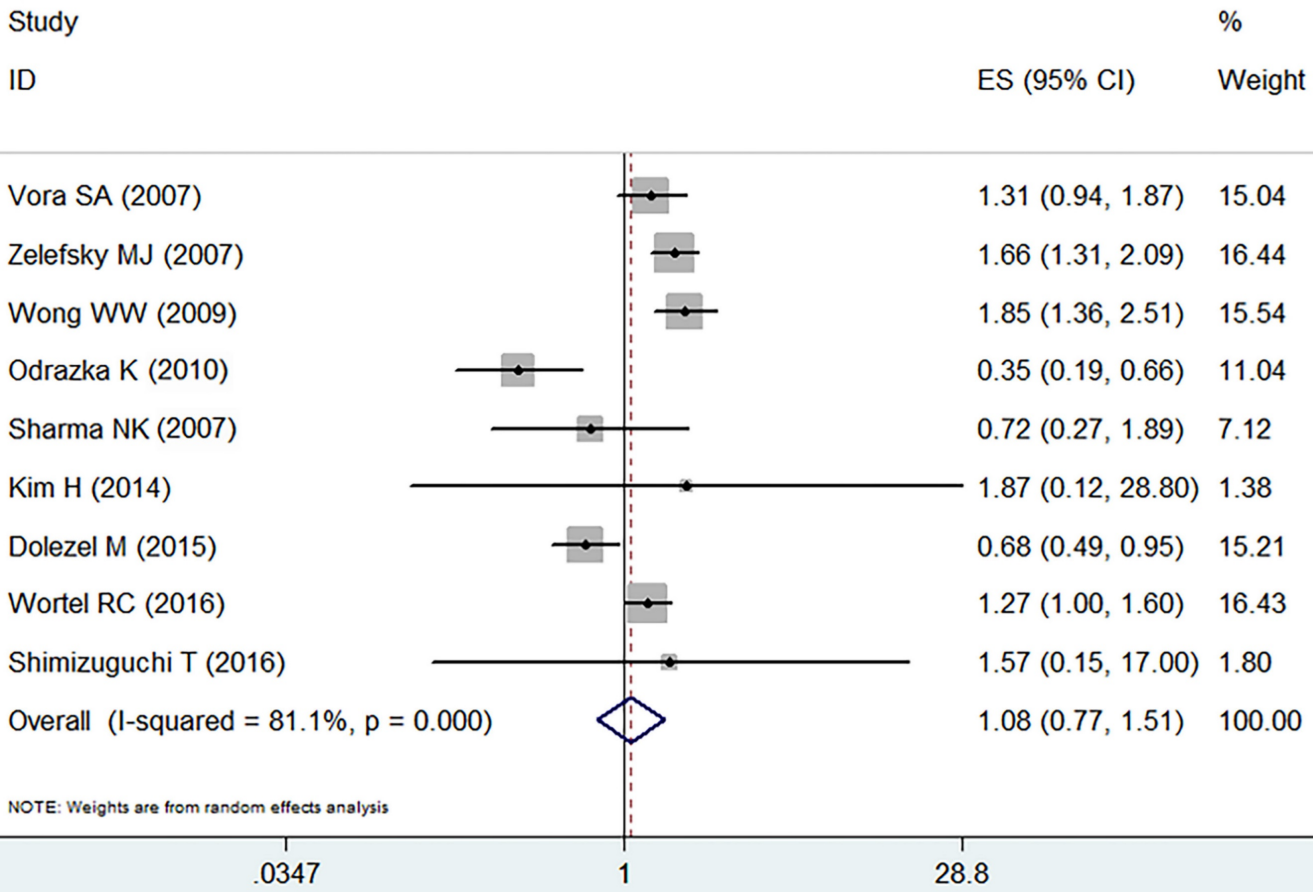
Forest plot for late GU adverse event.

With obvious heterogeneity found, the random effect model was employed (I^2^=81.1%, *p*=0.000). In those included studies, there was no significant difference between IMRT and 3D-CRT in grade 2-4 late GU adverse event (RR =1.08, 95% CI: 0.77-1.51, *p*=0.65,** Figure 5**).

### Biochemical control

**Figure 6:**
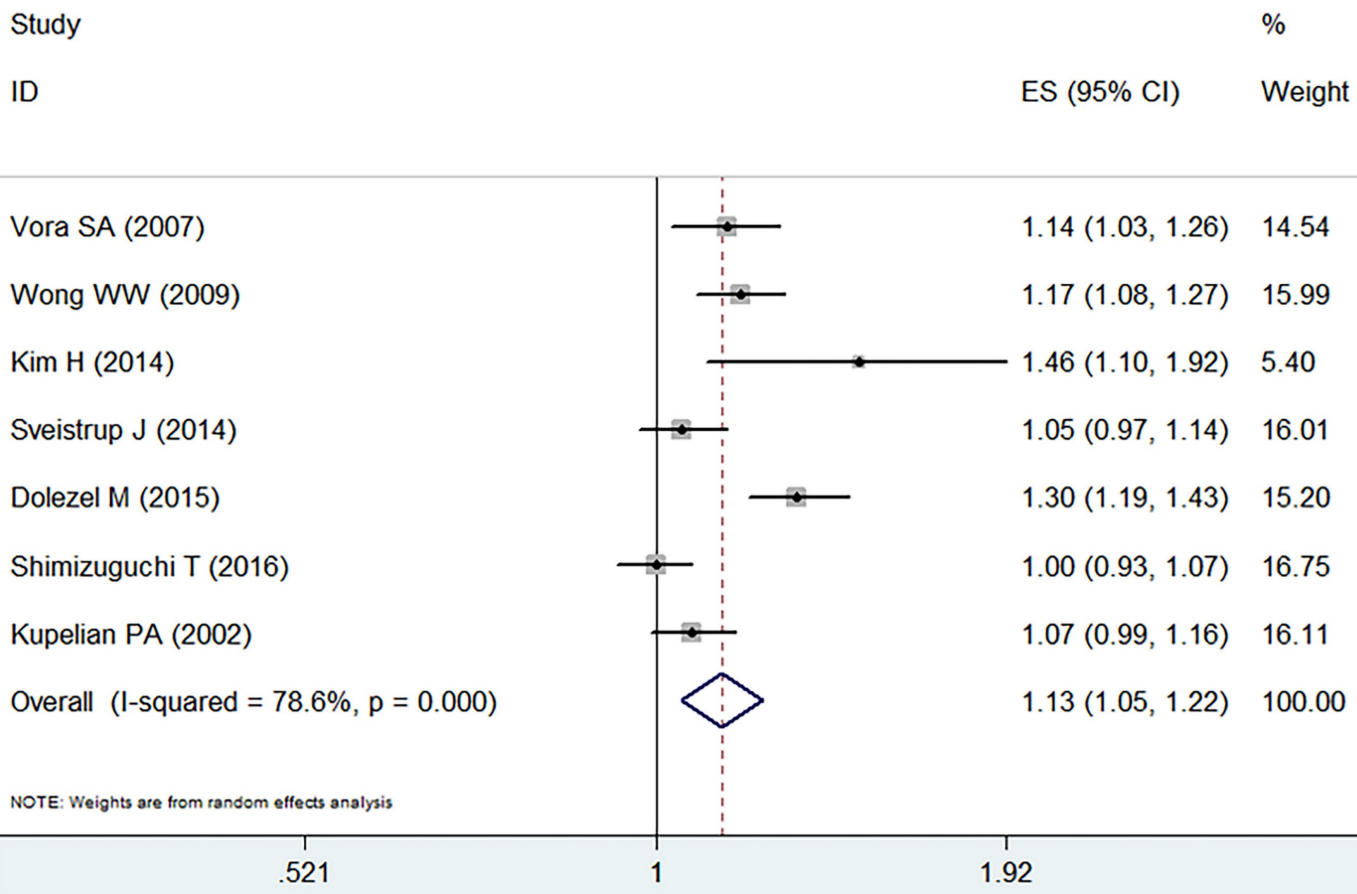
Forest plot for biochemical control.

There was a significant difference in biochemical control favoring IMRT (RR =1.13, 95% CI: 1.05-1.22, *p*=0.002,** Figure 6**). IMRT showed modest increase in biochemical control in comparison with 3D-CRT. Random effect model was employed because of the significant heterogeneity (I^2^=78.6%, *p*=0.000).

### Subgroup Analysis

The results of subgroup analysis (based on sample size, median follow-up time, PTV scope and study design) are presented in **Table 2-5**. In terms of “PTV scope,” IMRT can significantly reduce acute and late GI adverse events with a large PTV scope (Prostatic bed, pelvic nodes and seminal vesicles) (Acute: RR = 0.34, 95% CI: 0.17-0.69, *p*= 0.003; Late: RR = 0.46, 95% CI: 0.25-0.85,* p*= 0.013). However, IMRT failed to reduce acute and late GU adverse events with a large PTV scope (Acute: RR = 1.15, 95% CI: 0.86-1.53, *p*= 0.352; Late: RR = 0.80, 95% CI: 0.32-2.00,* p*= 0.636). For large sample size studies (n>100), IMRT can significantly reduce acute GI and GU adverse events (Acute GI: RR = 0.62, 95% CI: 0.46-0.84, *p*= 0.002; Acute GU: RR = 1.12, 95% CI: 1.03-1.22,* p*= 0.006).

### Sensitivity analysis and publication bias

**Figure 7:**
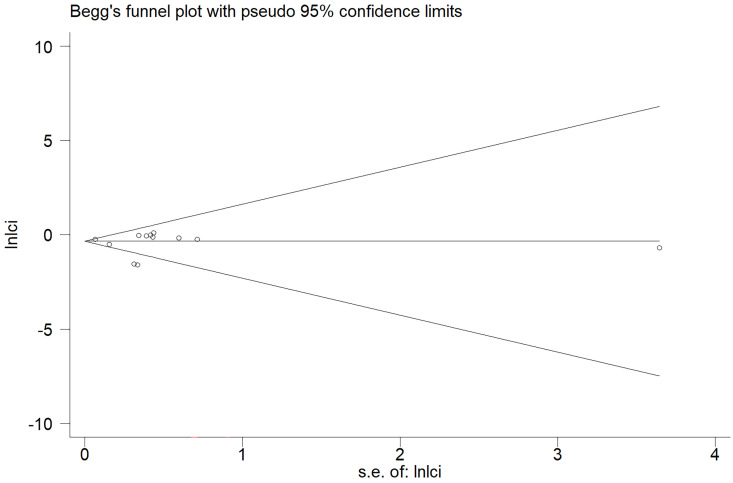
Funnel plots evaluating acute GU adverse event.

**Figure 8:**
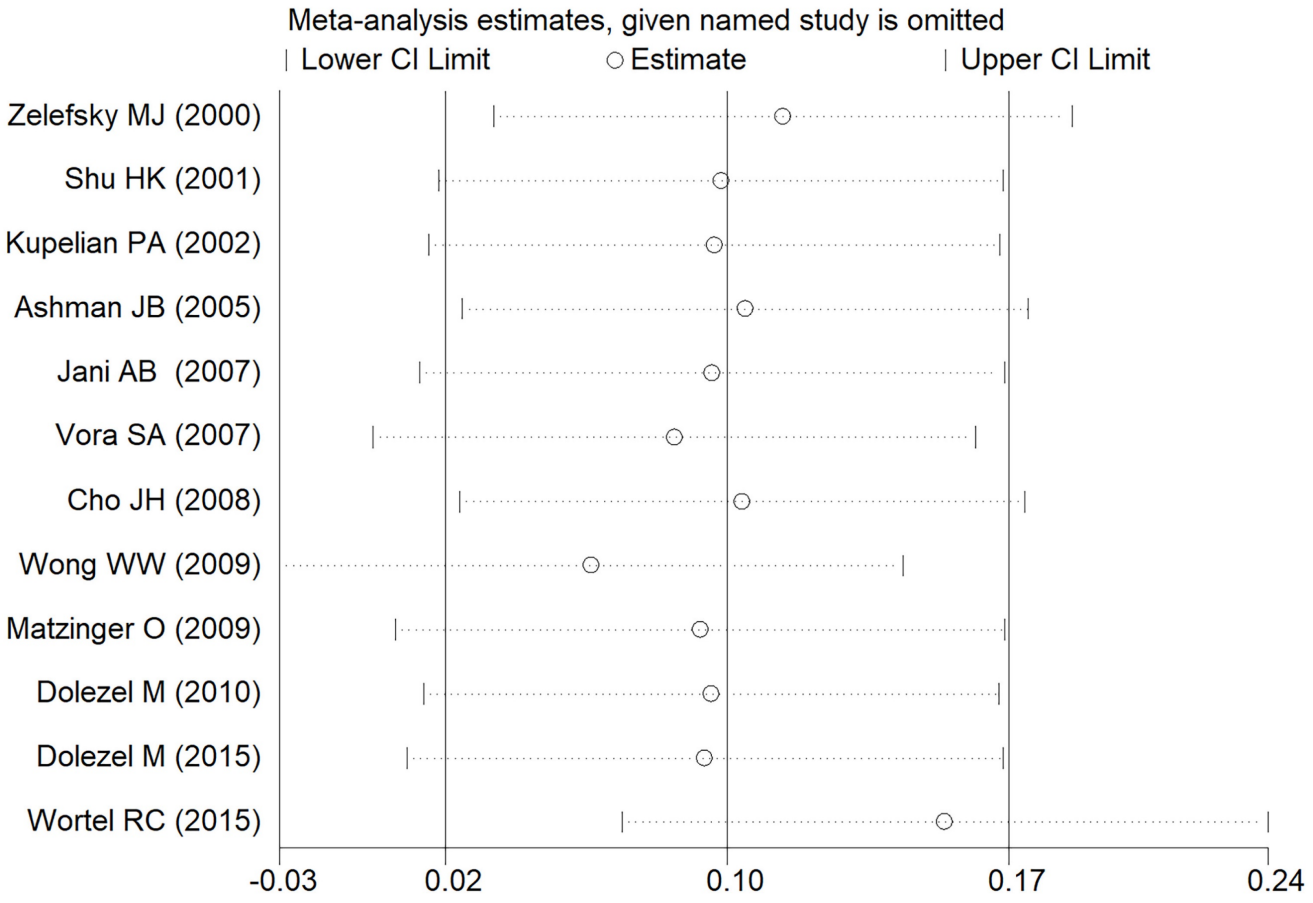
Sensitivity analysis of the acute GU adverse event.

In the meta-analysis of all outcomes, the Begg's funnel plot did not indicate any statistically significant asymmetry (**Figure 7**). A sensitivity analysis was carried out to make sure the robustness of the meta-analysis outcome. A statistically stable result was revealed by the sensitivity analysis's findings, which showed that none of the individual studies significantly affected the pooled HR or RR (**Figure 8**).

## Discussion

Twenty pertinent articles that evaluated the clinical results of PCa patients who underwent either IMRT or 3D-CRT were included in our meta-analysis. Our study's findings demonstrated that, as contrasted with 3D-CRT, IMRT was linked with a lower incidence of grade ≥ 2 acute GI adverse event and a higher BC. However, when compared to 3D-CRT, IMRT marginally increased the incidence of grade ≥ 2 acute GU adverse event while having identical grade ≥ 2 late GU adverse event. Furthermore, there were no appreciable variations in grade ≥ 2 late GI adverse event across the two treatment regimes. These data imply that IMRT, which has fewer side effects and enhanced PSA relapse-free survival, may be a better therapy option than 3D-CRT for patients with PCa.

Throughout the 1990s, numerous clinical trials proved the safety and efficacy of escalated-dose radiation for the treatment of localized PCa. However, it has been observed that increasing the prescribed dose is linked to an increased risk of late toxicities 40, 41. As a result, there was a desire for the development and implementation of highly conformal dose delivery systems in order to reduce these toxicities. Treatment technology advanced at the same time, with 3D-CRT replacing two-dimensional treatment 42. IMRT originated as an evolving version of 3D-CRT during the end of the 1990s 43. IMRT, a relatively new radiation therapy method, is capable of delivering a dose distribution across a complicated and irregular target volume. Planning studies have shown that IMRT can reduce the dosage to adjacent tissue while maintaining planning target volume coverage 44, 45.

However, there are several drawbacks to IMRT. When compared to 3D-CRT, IMRT exposes a greater volume of healthy tissues to modest doses of radiation, which may increase the risk of second malignancies. However, more complete and clear data are needed to determine the clinical significance of this issue 46. Furthermore, IMRT is a complex radiation treatment that necessitates a longer delivery time and greater physicist knowledge 47. IMRT is anticipated to cost around £1100 more than 3D-CRT, owing to more staff time for radiographers, medical experts, and physicists 48. Regardless, it is critical to assess the cost-effectiveness of IMRT, which may result in more quality-adjusted life-years (QALYs) at a lower overall cost 48. As a result, it is critical to carefully balance the benefits and dangers of IMRT.

GI and GU adverse events have considerably influenced the quality of life of PCa patients who have received radiation in all published trials. IMRT, according to Worter et al. 35, has significantly reduced acute GU and GI adverse effects. As the study advances, IMRT offers certain advantages in terms of reducing intestine damage when compared to 3D-CRT, but there is no significant difference in urinary tract harm^36^. According to our findings, IMRT is associated with a decrease in grade 2 acute GI adverse event and a little rise in grade ≥ 2 acute GU adverse event. However, no significant differences in grade ≥ 2 late GI and GU adverse events were found. This finding is supported by a study conducted by Yu et al 49, who found that both IMRT and 3D-CRT groups had a decreased incidence of grade ≥ 2 acute GI adverse event and a slightly higher morbidity of grade ≥ 2 acute GU adverse event. Fang and colleagues described observation of 94 patients who underwent IMRT. 13.8% of them suffered from Grade 2 acute GI toxicity while 28.7% experienced that level of GU toxicity. They stated that hypertension increases the risk of acute GI toxicity and ADT, similarly to International Prostate Symptom Score (IPSS), increases acute GU toxicity 50. In our study, the patients who underwent IMRT received ADT more than 3D-CRT. It might lead to an increase of acute GU adverse event. Grün et al found that the patients who were not able to maintain a partially filled bladder throughout treatment had a significantly higher risk of developing ≥ grade 2 GU acute adverse event 51. Like other clinical trials there did not appear to be any difference in late genitourinary toxicity by radiation technique 38. This may be related to the fact that the bladder neck and prostate urethra of 3DCRT and IMRT are inevitably part of the target volume. In addition, variable bladder filling makes the development of models of partial organ irradiation complex. Finally, the expression of late GU adverse event typically is years later than with GI adverse event and our follow-up is too short to identify any latent differences.

Numerous investigations have found that increasing the dose is associated with better biochemical and overall survival outcomes 4-7. IMRT appears to improve long-term survival in high-risk PCa patients without incurring more adverse events, as opposed to 3D-CRT 52. Our BC study findings also show a strong rising trend in IMRT when compared to 3D-CRT. Furthermore, following a long period of follow-up, the biochemical recurrence-free survival rate of PCa patients treated with 3D-CRT was significantly lower than that of IMRT-treated patients, particularly in patients with intermediate- to high-risk localized PCa 23, 33.

Aside from the inherent limitations of meta-analyses, our study had several significant drawbacks. For starters, our meta-analysis included a large number of retrospective studies, which may have caused bias when pooling the data. A bigger number of well-designed clinical trials and high-quality prospective studies should be conducted to further validate the findings. Furthermore, because the majority of the patients in our meta-analysis were Caucasian, caution should be exercised when extending the findings of this study to other ethnic communities.

In conclusion, our study found that IMRT is linked with a decrease in grade ≥ 2 acute GI adverse event and an improvement in biochemical control when compared to 3D-CRT, but a little rise in grade ≥ 2 acute GU adverse event. These findings show that IMRT should be considered as the best therapeutic approach for PCa. However, additional randomized controlled trials are required to determine the exact subset of PCa patients who might benefit the most from this treatment.

## Supplementary Material

Supplementary tables and information.

## Figures and Tables

**Table 1:** Study characteristics.

Study	Ref.	Year	Study design	Median follow-up (m) (3DCRT/ IMRT)	Number (3DCRT/IMRT)	Total dose/fraction dose (Gy) (3DCRT VS IMRT)	PTV	TNM or risk group	Score criteria	ADT% (3DCRT/IMRT)	Outcomes
Ashman JB	20	2005	Retro.	30/30	27 (14/13)	75.6/1.8 VS 81/1.8	Prostatic bed, pelvic nodes, seminal vesicles	T1c-T4	RTOG toxicity scale	100/100	Acute GI, GU
Cho JH	21	2008	Retro.	3/3	50 (35/15)	70.2/1.8 VS70/2.5	Prostatic bed	T1-T4N0-1	RTOG toxicity scale	44/44	Acute GI, GU
Dolezel M	22	2010	Pro.	68.4/37.2	232 (94/138)	74/2 VS78/2	Prostatic bed, pelvic nodes, seminal vesicles	T1-T3 All risk group	RTOG toxicity scale	94.7/55	Acute GI, GU
Dolezel M	23	2015	Pro.	104/60	533 (320/233)	70-74/2 VS 78-82/2	Prostatic bed, seminal vesicles	Localized prostate cancer	RTOG toxicity scale, ASTRO Phoenix definitioncity	40.3/62.3	Acute GI, GU Late GI, GU BC
Jani AB	24	2007	Pro.	NR/NR	481(373/108)	68.5/1.8-2 VS 75/1.8-2	Prostatic bed, seminal vesicles	T1-T4	RTOG toxicity scale	53/51	Acute GI, GU
Kim H	25	2014	Retro.	78.6/73.4	86 (56/30)	70/1.8 VS 70/2.5	Prostatic bed, pelvic nodes, seminal vesicles	T1-T3bN0-N1	RTOG toxicity scale	56.7/53.6	Late GI, GU BC
Kupelian PA	26	2002	Retro.	25/25	282(116/166)	78/2 VS 70/2.5	Prostatic bed, pelvic nodes, seminal vesicles	T1-T3	RTOG toxicity scale, ASTRO Phoenix definitioncity	72/60	Acute GU, BC
Odrazka K	27	2010	Retro.	70.8/36	340(228/112)	70/2 VS 78/2	Prostatic bed, seminal vesicles	T1-3N0(pN0)M0	RTOG toxicity scale	19.7/54.5	Late GU
Sharma NK	28	2007	Retro.	86/40	293(170/123)	76/2 VS 76/1.8	Prostatic bed, pelvic nodes, seminal vesicles	T1-T3Nx-N0M0	RTOG toxicity scale	100/100	Late GI, GU
Shimizuguchi T	29	2016	Retro.	61.2/54	159(70/89)	76/2 VS 76/2	Prostatic bed, seminal vesicles	T1-T3N0M0	CTCAE version 4.0	88/90	Late GI, GU BC
Shu HK	30	2001	Retro.	30.1/18.7	44(26/18)	NR	Prostatic bed, seminal vesicles	T1-T3	RTOG toxicity scale	79.5	Acute GI, GU
Sveistrup J	31	2014	Retro.	98.4/42	503(115/388)	76/2 VS 78/2	Prostatic bed, seminal vesicles	High risk	CTCAE version 4.0, ASTRO Phoenix definitioncity	88/95	BC
Troeller A	32	2014	Pro.	106.8/55.2	1115(457/658)	75.6/1.8 VS 75.6/1.8	Prostatic bed, seminal vesicles	NR	CTCAE version 3.0	23.2/19.9	Late GI
Vora SA	33	2007	Retro.	60/48	416(271/145)	68.4/NR VS 75.6/NR	Prostatic bed, seminal vesicles	T1b-T3b	RTOG toxicity scale, ASTRO Phoenix definitioncity	17.6/30.3	Acute GI, GU Late GI, GU BC
Wong WW	34	2009	Retro.	120/120	584(270/314)	68.4/1.8-2 VS 75.6/NR	Prostatic bed, seminal vesicles	T1c-T3N0M0	RTOG toxicity scale, ASTRO Phoenix definitioncity	17/36	Acute GI, GU Late GI, GU BC
Wortel RC	35	2015	RCT	3/3	475(215/260)	78/2 VS 78/2	Prostatic bed, seminal vesicles	T1-T4	RTOG toxicity scale	19.5/66.9	Acute GI, GU
Wortel RC	36	2016	RCT	62/57	431(189/242)	78/2 VS 78/2	Prostatic bed, seminal vesicles	T1b-T4Nx-0Mx-0	RTOG toxicity scale	40/66	Late GI, GU
Zelefsky MJ	37	2000	Retro.	39/12	232(61/171)	81/1.8 VS 81/1.8	Prostatic bed, seminal vesicles	T1c-T3	RTOG toxicity scale	34/53	Acute GU
Zelefsky MJ	38	2008	Retro.	120/78	1571(830/741)	66-81/1.8 VS 81/NR	NR	T1-T3	CTCAE version 3.0	43	Late GU
Matzinger O	39	2009	RCT	NR/NR	791(652/139)	70-78/2 VS 74- 78/2	NR	cT1b-2cN0M0	CTCAE version 2.0	50	Acute GI, GU

BC: Biochemical control; PCa: Prostate cancer; IMRT: Intensity-modulated radiation therapy; 3D-CRT: Three-dimensional conformal radiotherapy; RTOG: Radiation Therapy Oncology Group; GI: gastrointestinal; GU: Genitourinary; RCT: Randomized clinical trials; Retro.: Retrospective study; Pro.: Prospective study; PTV: Planning target volume; NR: Not reported; Ref.: References; ADT: Androgen deprivation therapy. CTCAE: Common Terminology Criteria of Adverse Events.

**Table 2:** Subgroup analysis of Acute GI

Items		No. of studies	RR	95% CI	P value
Sample size	≤100	3	0.64	0.07-5.74	0.692
	>100	7	0.62	0.46-0.84	0.002
Study design	Retrospective study	5	0.87	0.66-1.15	0.333
	Prospective	3	0.40	0.24-0.67	0.001
	RCT	2	0.74	0.41-1.35	0.330
Median follow-up(m)	<60	6	0.61	0.38-0.96	0.035
	≥60	2	0.48	0.16-1.49	0.206
	NR	2	0.81	0.50-1.31	0.382
PTV scope	Prostatic bed and seminal vesicles	6	0.65	0.45-0.93	0.017
	Prostatic bed, pelvic nodes and seminal vesicles	2	0.34	0.17-0.69	0.003
	Prostatic bed	1	0.39	0.05-3.00	0.366

RCT: Randomized clinical trials; NR: Not reported; PTV: Planning target volume; RR: Risk ratio

**Table 3:** Subgroup analysis of Acute GU

Items		No. of studies	RR	95% CI	P value
Sample size	≤100	5	0.93	0.73-1.19	0.568
	>100	7	1.12	1.03-1.22	0.006
Study design	Retrospective study	7	1.17	1.04-1.33	0.011
	Prospective	3	1.17	0.99-1.38	0.06
	RCT	2	0.99	0.88-1.13	0.927
Median follow-up(m)	<60	8	1.00	0.90-1.12	0.976
	≥60	2	1.25	1.08-1.44	0.003
	NR	2	1.15	0.98-1.36	0.091
PTV scope	Prostatic bed and seminal vesicles	7	1.09	1.00-1.19	0.047
	Prostatic bed, pelvic nodes and seminal vesicles	3	1.15	0.86-1.53	0.352
	Prostatic bed	1	0.58	0.22-1.53	0.273

RCT: Randomized clinical trials; NR: Not reported; PTV: Planning target volume; RR: Risk ratio

**Table 4:** Subgroup analysis of Late GI

Items		No. of studies	RR	95% CI	P value
Sample size	≤100	2	0.44	0.13-1.52	0.195
	>100	6	0.66	0.36-1.22	0.186
Study design	Retrospective study	5	0.74	0.42-1.32	0.310
	Prospective	2	0.48	0.14-1.59	0.229
	RCT	1	0.66	0.50-0.88	0.004
Median follow-up(m)	<60	5	0.51	0.24-1.08	0.078
	≥60	3	0.89	0.70-1.12	0.321
PTV scope	Prostatic bed and seminal vesicles	6	0.64	0.34-1.23	0.181
	Prostatic bed, pelvic nodes and seminal vesicles	2	0.46	0.25-0.85	0.013

RCT: Randomized clinical trials; PTV: Planning target volume; RR: Risk ratio

**Table 5:** Subgroup analysis of Late GU

Items		No. of studies	RR	95% CI	P value
Sample size	≤100	2	1.69	0.28-10.14	0.565
	>100	7	1.06	0.75-1.50	0.737
Study design	Retrospective study	7	1.14	0.75-1.75	0.531
	Prospective	1	0.68	0.49-0.95	0.022
	RCT	1	1.27	1.00-1.61	0.046
Median follow-up(m)	<60	5	0.90	0.55-1.47	0.670
	≥60	4	1.30	0.76-2.25	0.340
PTV scope	Prostatic bed and seminal vesicles	6	1.10	0.77-1.58	0.585
	Prostatic bed, pelvic nodes and seminal vesicles	2	0.80	0.32-2.00	0.636

RCT: Randomized clinical trials; PTV: Planning target volume; RR: Risk ratio

## Abbreviations

IMRT: Intensity modulated radiation therapy
3D-CRT: Three-dimensional conformal radiation therapy
PCa: Prostate cancer
GI: Gastrointestinal
GU: Genitourinary
HR: Hazard ratio
RR: Risk ratio
PRISMA: Preferred Reporting Items for Systematic Reviews and Meta-analyses guidelines
CI: Confidence interval
PTV: Planning target volume
QALYs: Quality-adjusted life-years
NOS: Newcastle-Ottawa quality assessment scale
TNM: Tumor-node-metastasis
CTCAE: Common Terminology Criteria of Adverse
RTOG: Radiation Therapy Oncology Group
